# Evaluating the Potential Effectiveness of Compensatory Mitigation Strategies for Marine Bycatch

**DOI:** 10.1371/journal.pone.0002480

**Published:** 2008-06-18

**Authors:** Myra Finkelstein, Victoria Bakker, Daniel F. Doak, Ben Sullivan, Rebecca Lewison, William H. Satterthwaite, Peter B. McIntyre, Shaye Wolf, David Priddel, Jennifer M. Arnold, Robert W. Henry, Paul Sievert, John Croxall

**Affiliations:** 1 Department of Ecology and Evolutionary Biology, University of California Santa Cruz, Santa Cruz, California, United States of America; 2 Department of Zoology and Physiology, University of Wyoming, Laramie, Wyoming, United States of America; 3 C/Australian Antarctic Division, BirdLife International Global Seabird Programme, Kingston, Tasmania, Australia; 4 Biology Department, San Diego State University, San Diego, California, United States of America; 5 Applied Math and Statistics, University of California Santa Cruz, Santa Cruz, California, United States of America; 6 School of Natural Resources and Environment, University of Michigan, Ann Arbor, Michigan, United States of America; 7 Center for Biological Diversity, San Francisco, California, United States of America; 8 Department of Environment and Climate Change (New South Wales), Hurstville, New South Wales, Australia; 9 Division of Science, Pennsylvania State University, Reading, Pennsylvania, United States of America; 10 United States Geological Survey, Massachusetts Cooperative Fish & Wildlife Research Unit, University of Massachusetts Amherst, Amherst, Massachusetts, United States of America; 11 BirdLife International Global Seabird Programme, Girton, Cambridge, United Kingdom; University of Aberdeen, United Kingdom

## Abstract

Conservationists are continually seeking new strategies to reverse population declines and safeguard against species extinctions. Here we evaluate the potential efficacy of a recently proposed approach to offset a major anthropogenic threat to many marine vertebrates: incidental bycatch in commercial fisheries operations. This new approach, compensatory mitigation for marine bycatch (CMMB), is conceived as a way to replace or reduce mandated restrictions on fishing activities with compensatory activities (e.g., removal of introduced predators from islands) funded by levies placed on fishers. While efforts are underway to bring CMMB into policy discussions, to date there has not been a detailed evaluation of CMMB's potential as a conservation tool, and in particular, a list of necessary and sufficient criteria that CMMB must meet to be an effective conservation strategy. Here we present a list of criteria to assess CMMB that are tied to critical ecological aspects of the species targeted for conservation, the range of possible mitigation activities, and the multi-species impact of fisheries bycatch. We conclude that, overall, CMMB has little potential for benefit and a substantial potential for harm if implemented to solve most fisheries bycatch problems. In particular, CMMB is likely to be effective only when applied to short-lived and highly-fecund species (not the characteristics of most bycatch-impacted species) and to fisheries that take few non-target species, and especially few non-seabird species (not the characteristics of most fisheries). Thus, CMMB appears to have limited application and should only be implemented after rigorous appraisal on a case-specific basis; otherwise it has the potential to accelerate declines of marine species currently threatened by fisheries bycatch.

## Introduction

**Figure 1:**
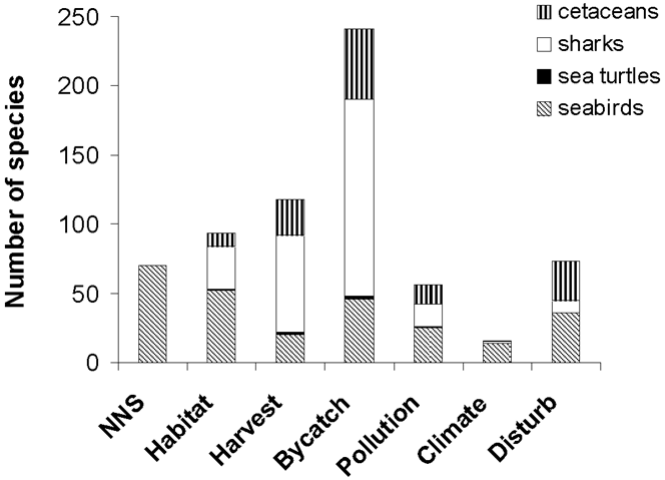
Bycatch is the principal threat to at-risk marine vertebrates. Total number of species of cetaceans, sharks, sea turtles, and seabirds in the IUCN red list database (http://www.iucnredlist.org/) affected by the following threat categories: NNS = invasive non-native species; Habitat = habitat destruction and degradation; Harvest = harvesting; Bycatch = accidental mortality from fisheries bycatch; Pollution = pollution (land and water); Climate = pollution relating to the atmosphere and climate change; Disturb = human disturbance, persecution, noise pollution, and collisions (from Doak et al. [66]). Although not listed as a primary threat in the IUCN database, non-native species are also known to impact sea turtles on their breeding grounds [67].

One of the most vexing and current crises in marine conservation is the inadvertent and unsustainable catch of non-target marine species (i.e., bycatch) in commercial fisheries. Bycatch is increasingly recognized as one of the principal threats to many marine vertebrates, including multiple species of sharks, sea turtles, and seabirds [1]–[4]. Indeed, more marine vertebrates are threatened by bycatch (239 species) than by any other major hazard, including non-native species (70 species) or targeted harvesting (118 species) (http://www.iucnredlist.org/, Figure 1). In response to increased recognition that marine bycatch causes dramatic population declines, various agencies worldwide have mandated changes to fishing gear and spatial or temporal closures of fisheries to reduce bycatch mortality. Together, these mitigation actions have yielded considerable reductions in bycatch of at least some threatened species [5]. While this progress in reducing bycatch towards sustainable levels is encouraging, more effective management approaches to address this global problem are clearly needed.

Wilcox and Donlan (henceforth W&D) recently proposed a new approach to marine conservation that they suggest would significantly improve the protection of sensitive marine species impacted by fisheries bycatch [6]. To date, management of bycatch threats have focused on modifying fishing practices to reduce bycatch (e.g., seasonal closures, turtle exclusion devices, weighted hooks, circle hooks) or, as an emergency measure, closing or curtailing fisheries [7]–[9]. W&D argue that closing or curtailing fisheries to reduce bycatch is a poor strategy because “return on investment” is low: many fisheries are enormously lucrative and this economic gain must be restricted to reduce bycatch mortality. As an alternative, they suggest that an economically efficient way to conserve populations affected by bycatch is to impose a bycatch levy on fishers to fund compensatory mitigation activities that target threats at locations where affected marine populations breed. A similar compensatory approach has been applied with mixed results to compensate for (or offset) other environmentally damaging activities, including land degradation due to filling of wetlands [10] and, more recently, the “carbon footprints” of individuals, organizations, and rock band tours (www.nativeenergy.com). Compensatory mitigation for marine bycatch (CMMB) is thus the application of an established, albeit controversial [11], [12], strategy applied in the novel setting of fisheries bycatch.

W&D have offered a broad outline of how CMMB might function, but provided few details and described only one quantitative case study, which they conducted for the flesh-footed shearwater (FFSH, *Puffinus carneipes*) on Lord Howe Island off mainland Australia. Because a new approach to solving marine bycatch could have dramatic conservation implications, a more careful analysis of CMMB, and in particular, a thorough consideration of the necessary criteria for this approach to yield substantial and reliable conservation benefits, is essential.

Here, we provide an analysis of the prerequisites that CMMB would need to meet in order to be considered a biologically promising solution for any particular bycatch situation. We focus on the ecological aspects of the species targeted by CMMB, the range of possible mitigation measures, and the multi-species impact of fisheries bycatch. Although this review does not extend to economic criteria, any viable strategy for solving conservation problems must first succeed from a biological perspective, and at a minimum, must be capable of averting population declines, regardless of economic costs and benefits. Thus, we explore the key criteria to meet the ecological goal of reversing population declines and encourage economists to assess the economic aspects of biologically viable proposals.

## Analysis

### What is Compensatory Mitigation for Marine Bycatch (CMMB)?

Compensatory mitigation strategies are based on the premise that damage caused in one location or time can be offset by beneficial activities elsewhere. For example, destruction of a wetland area during construction of a deep-water channel is compensated for by restoration of a degraded wetland area somewhere else. This logic of redressing harm through compensation, rather than by reducing it directly, is pivotal to CMMB and is the foundation of its allure. While a CMMB program could be combined with continued use of mitigation strategies that have little cost or effect on the catch rates of fisheries (e.g., streamer lines to reduce seabird bycatch), CMMB is not a proposal to add a new mitigation measure with substantial costs (e.g., halting or reducing fishing effort, and hence reducing catch) to existing regulatory controls on fisheries. Instead, CMMB is a proposal to continue or expand current levels of fishing effort, and thus current bycatch levels, by lessening restrictions on fishing effort in exchange for fees that would fund indirect compensatory mitigation measures.

W&D illustrated the use of CMMB with a case study of the FFSH on Lord Howe Island, one of their numerous breeding colonies. The FFSH population is declining, in part due to high mortality (recently as high as ∼3700 individuals/year) on the longline hooks of the Eastern Tuna and Billfish Fishery (ETBF) [13], [14]. Through CMMB, fees on longline vessels in the ETBF would be used to fund eradication of introduced rats on Lord Howe Island, which W&D suggest are major predators of FFSH eggs and chicks. W&D present a population model that they use to argue that rat removal would have benefits for shearwater populations that exceed the detrimental impact of continued bycatch mortality (but see ***Re-Analysis of a Case Study*** below and Supplement S1). As with this case study of the FFSH, to date CMMB examples have proposed the substitution of terrestrial mitigation actions that benefit reproductive output on the breeding grounds for continuing adult (and subadult) mortality due to fisheries bycatch [6], [15].

### Suggested Criteria for CMMB to be Effective as a Conservation Strategy

CMMB offers the potential to substitute a politically difficult management approach to curtailing the number of threatened species killed as fisheries bycatch (e.g., direct reduction of bycatch rates) with alternate management methods (e.g., eradication of introduced species on islands). However, before such a substitution of one management effort with another is applied to a particular fishery, conservation biologists and managers must have some evidence that the compensatory strategy can yield positive outcomes and not result in a net conservation loss. We present five criteria to serve as a systematic way to assess the potential of CMMB as a tool for creating ‘bycatch neutral’ fisheries, as recent media coverage has labeled the CMMB approach (e.g., http://www.smithsonianmag.com/specialsections/ecocenter/bycatch.html):

1. ***Mitigation actions must have a realistic potential of fully compensating for bycatch impacts on population growth***
2. ***Proven and successful conservation activities must exist for a bycatch-impacted species***
3. ***The spatial scales of mitigation benefits and bycatch impacts must be comparable when assessing the effects of CMMB on population growth***
4. ***CMMB must account for the potential indirect effects of fisheries incentives and fees on bycatch rates***
5. ***CMMB for species meeting criteria 1 through 4 should not increase bycatch impacts to other at-risk species without adequate compensation***

### 1) Mitigation actions must have a realistic potential of fully compensating for bycatch impacts on population growth

In order for CMMB to meet the long-term goals of resource managers and conservationists, the requirements of international laws, and the mandates of most fisheries management agencies, it must offset bycatch impacts to the point that population growth – even slight growth – is possible. In other words, the net result of the negative effects of bycatch and the positive effects of a designated mitigation activity on population growth must result in a positive, or at least stable, population growth trajectory. Otherwise, compensatory mitigation will be ineffective at reversing, and may even accelerate, the trajectories to extinction of many marine species. Meeting this criterion requires the use of quantitative population analysis and some agreed upon standard of population health. We concur with W&D that population growth rate is a reasonable metric, however for critically endangered species, changes in short-term stochastic risk of extinction may be a more sensitive and meaningful measure of a CMMB program's success [16] and for many harvested species, simple deterministic growth rates may poorly reflect real population dynamics [17].

Although the continued survival of threatened populations is so basic a criterion that it may seem unnecessary to discuss, it is indeed a major stumbling block for a successful CMMB program. As we note above, examples of possible CMMB programs use enhancement of early life stage survival to balance mortality of older aged individuals in fisheries. Most species identified as critically endangered by bycatch are moderately to very long-lived, including large sharks, seabirds, and sea turtles. The long life spans of most threatened marine vertebrates are combined with delayed reproductive maturity and/or low reproductive rates; these life history traits create the *prima facie* conditions that make these species at-risk because they sharply limit their maximum attainable rates of population growth. In a nutshell, the problem is the relative reproductive value of different life history stages: for species with long pre-reproductive periods, delayed senescence and low to moderate fecundity, the effect on population growth of losing each adult must be offset by saving dozens to hundreds of young animals. For example, 588 hatchling loggerhead sea turtles (*Caretta caretta*) would have to be saved to equal the mortality of one adult in terms of contribution to future population growth [18]. Thus, balancing adult bycatch mortality with increases in other demographic rates is biologically difficult or impossible as these species exhibit extreme sensitivity to elevated mortality in older age classes [18]–[21].

Given the typical long-lived, slow-maturing life history of most species threatened by bycatch (seabirds, sea turtles, sharks, cetaceans), CMMB efforts that increase survival of offspring to compensate for mortality of adults are unlikely to reverse population declines. One of the first and best analyses of this problem – and one that is literally now a textbook example – is for loggerhead sea turtles impacted by bycatch in shrimp trawlers [18], [22]. As Crouse et al. [18] showed, 100% protection of eggs and hatchlings on beaches (exactly the type of mitigation activity that CMMB could feasibly fund) would have only a miniscule impact on reversing population declines, while even moderate decreases in bycatch of older animals would switch the population trajectory from declining to increasing. Numerous demographic assessments show that for most long-lived marine species, adult survival is paramount for population growth [23]–[25]. For these species, terrestrial mitigation measures must be exceptionally effective to counteract the effects of low-level bycatch mortality of adults; existing research suggests high bycatch rates of older age classes simply cannot be balanced by mitigation targeting younger animals.

### 2) Proven and successful conservation activities must exist for a bycatch-impacted species

Other types of mitigation approaches, such as mitigation banking of wetlands, include explicit acknowledgement of uncertainty about the possible outcomes of mitigation and restoration approaches [26]. With respect to CMMB, there is certainty as to the negative effects of bycatch, but uncertainty as to the efficacy of mitigation procedures. Given that fisheries bycatch is a demonstrable source of mortality for many long-lived marine species that are declining at rapid rates [1], [3], [4], we believe that CMMB programs must establish the effectiveness of proposed compensatory mitigation options *before* they are substituted as a bycatch conservation strategy.

The proposed uses of CMMB have emphasized the funding of land-based mitigation activities, in particular the removal of non-native species from islands [6], [15]. As discussed, the basic biology of most bycatch-impacted species makes it difficult to compensate for the effects of mortality on older age classes due to bycatch. Yet, an even more basic problem with assessing the widespread applicability of CMMB proposals is the lack of tested or even understood mitigation methods for many species.

**Figure 2:**
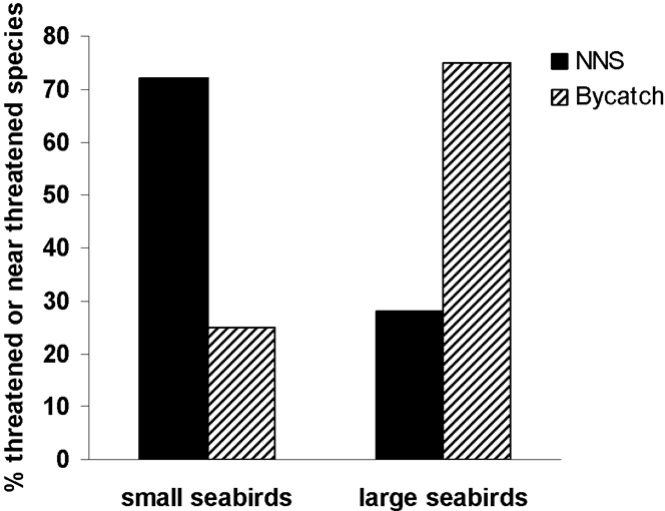
Bycatch is the main threat to imperiled large bodied seabirds. Percentage of IUCN listed Near Threatened and Threatened small (<600 g) and large (>600 g) seabird species impacted by non-native species (NNS, n = 21 for birds <600 g and 7 for birds >600 g) and fisheries bycatch (bycatch, n = 7 for birds <600 g and 18 for birds >600 g). Data on ‘Threat’ types classified by the IUCN Threats Authority File (see http://www.iucnredlist.org/info/major_threats) and held in BirdLife International's World Bird Database. Only threats scored as High or Medium impact were considered, where impact of threats is calculated from the sum of scores assigned for timing (past, continuing, future), scope (proportion of total population affected), and severity (rate of declines caused by the threat within the scope; see http://www.birdlife.org/datazone/species/terms/threats.html for details).

**Figure 3:**
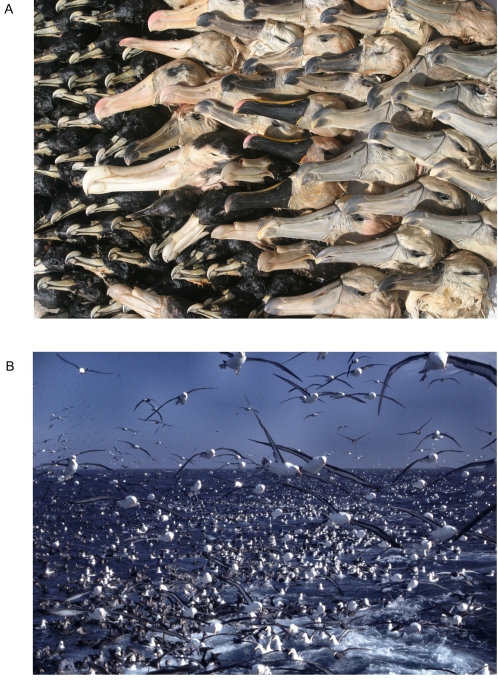
Bycatch impacts multiple seabird species. A) Albatross and other seabird bycatch from a pelagic longline fishing vessel operating in South Africa for a period of one month in 2005. Photo: Peter Ryan. B) Adult black-browed albatrosses and giant-petrels scavenging discards behind a trawler in the Falkland Islands. Photo: Ben Sullivan – Falklands Conservation.

W&D have highlighted exotic animal eradications on islands as a feasible and potentially beneficial use of CMMB funding for seabird bycatch solutions. However, for seabirds, a discrepancy exists between seabird species most affected by bycatch (large, >600 g) and those most impacted by non-native predators (small, <600 g; Figure 2) [27]. Thus, with a few exceptions (e.g., Cuthbert and Hilton [28]), eradicating non-native predators would not substantially help the suite of large-bodied seabirds most threatened by fisheries bycatch (Figure 3).

Furthermore, the majority of species threatened by bycatch are cetaceans and sharks (Figure 1). These species do not aggregate to breed in localized terrestrial sites where relatively inexpensive and effective mitigation activities could be employed, and for the vast majority, too little is understood of their early life stages to plan feasible mitigation activities of any kind. For example, even though some form of mitigation (e.g., restoration of estuarine spawning habitat) might benefit a few shark populations [29], the lack of basic data on life history and reproduction of many shark species precludes the widespread evaluation and use of this approach.

### 3) The spatial scales of mitigation benefits and bycatch impacts must be comparable when assessing the effects of CMMB on population growth

The most likely targets for terrestrial mitigation efforts under CMMB are seabirds and sea turtles [6] (www.advancedconservation.org/offset/). For these species, comparing the spatial scales at which bycatch and compensatory mitigation occur relative to the geographic range of the affected populations is critical. Industrial fisheries operate in marine ecosystems around the globe; therefore bycatch mortality typically affects susceptible species throughout large parts of their geographic ranges. In contrast, terrestrial threats rarely impact all breeding locations used by a given bird or turtle population.

The dynamics of many seabird populations currently approximate source-sink population structures [30]. In the absence of bycatch, the one or more breeding colonies that are affected by introduced predators or other threats on breeding islands are likely to have rates of population growth less than one – they are sinks. In contrast, colonies without these localized threats are sources, with net population increase and “export” of some progeny to sink colonies. The importance of source-sink dynamics lies in the difference between the sensitivity of overall population growth to changes in the dynamics of the source versus sink subpopulations. As a wide range of analyses of many source-sink situations has shown [30]–[32], overall population growth and extinction risk is far more sensitive to changes in the demography of the source than sink parts of the population. Indeed, unless there are extremely high rates of movement between source and sink areas, the overall population growth rate, extinction risk, and total population size of coupled source-sink populations are almost entirely determined by the vital rates of the source population. Source-sink dynamics is highly relevant to the CMMB strategy because it would be easy, without explicit consideration of the spatial population structure, to believe that bycatch mortality impacting all parts of the population could be successfully ‘mitigated’ by removing introduced predators on one or more sink colony. In fact, if bycatch effects are experienced by an entire network of subpopulations, localized mitigation actions on a single subpopulation will almost never be adequate to offset bycatch mortality.

An example of a source-sink population structure typical of many seabirds is illustrated by the white-chinned petrel (*Procellaria aequinoctialis*). The white-chinned petrel breeds on a small number of sub-Antarctic island groups, but is abundant and widely distributed throughout the southern oceans where it constitutes the majority of seabird bycatch in longline fisheries [33]. While rats can be significant predators on some white-chinned petrel colonies, approximately half of the petrel's breeding subpopulations do not have rats, and even on islands with rats, about half of the colonies are rat-free (e.g., South Georgia [34]). Thus, removal of non-native species at one location may boost a local subpopulation's viability, but bycatch would continue to drive the species toward extinction by killing older individuals from all populations. For this typical species, CMMB is unlikely to offset or reduce population declines.

**Figure 4:**
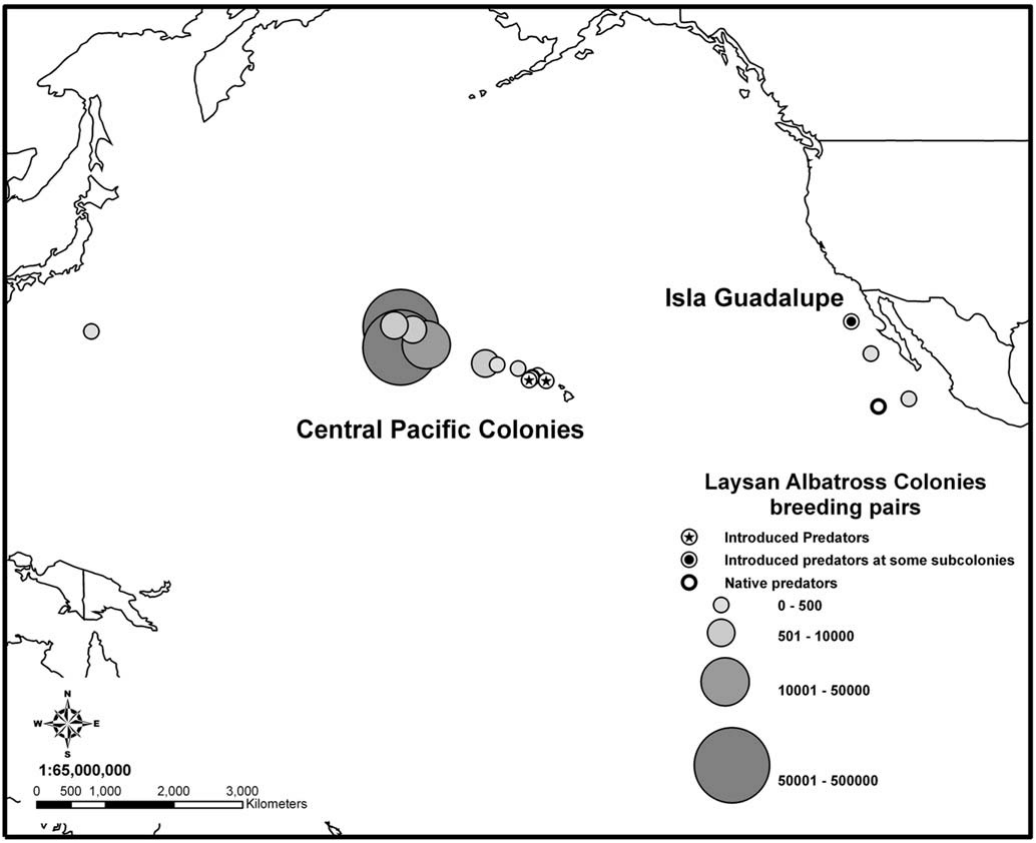
Global distribution of Laysan albatross (*Phoebastria immutabilis*) breeding colonies with and without island predators. The majority (>99%) of Laysan albatross breed on islands with no introduced predators (excluding the house mouse, *Mus musculus*) in the Hawaiian archipelago. Introduced predators (e.g., cats) affect Laysan albatross on a Guadalupe Island sub-colony as well as a few small (<500 pairs) colonies on the main Hawaiian Islands (data from U.S. Fish and Wildlife Service and R.W. Henry).

Another seabird example is the Laysan albatross (*Phoebastria immutabilis*), which breeds primarily in the northwestern Hawaiian Islands, United States of America, but also on Guadalupe Island, Mexico [35] (Figure 4). Laysan albatrosses are killed in fisheries bycatch throughout their range as well as by cats during the breeding season on Guadalupe Island (R.W. Henry, pers. obs.) [36]. Although cat mortality does impact the local albatross population on Guadalupe Island, the entire Guadalupe Island population comprises <0.02% of the worldwide population (approximately 400 of 2.5 million birds), with >99% breeding on islands with no known introduced predators (excluding the house mouse (*Mus musculus*), which is not known to prey on Laysan albatross, but see [28]) (Figure 4). Additionally, less than 13% of the Guadalupe population is impacted by cats; most of this population breeds on off-shore islets with no introduced predators (R.W. Henry, pers. obs.). Because the global population of Laysan albatross is believed to be affected by bycatch, cat eradication on Guadalupe Island is unlikely to be capable of offsetting the effect of bycatch mortality on overall Laysan albatross population growth.

The white-chinned petrel and Laysan albatross examples epitomize the common sense argument that when a population is distributed across a range of source and sink habitat areas, conservation planning must prioritize protection of current source subpopulations to ensure species survival [31], [37]. This argument is particularly relevant to seabirds and turtles by virtue of their nest site fidelity, where movement between breeding sites is extremely limited. In contrast to CMMB, other efforts such as wetlands banking avoid this problem because the spatial scales of damage and mitigation are equivalent. In the case of CMMB, careful consideration of the impacts of bycatch versus mitigation across a population network is essential to achieve an accurate understanding of the effectiveness of localized mitigation relative to the threat of bycatch mortality.

### 4) CMMB must account for the potential indirect effects of fisheries incentives and fees on bycatch rates

A detailed description of the mechanics of how CMMB would change fisheries bycatch rates also requires careful consideration. CMMB has been described as a fishery tax based on the total bycatch per vessel or fleet [6], [15]. Whether this tax scheme will result in bycatch reductions will be determined by the tax rate, to what species to the tax is applied, and how accurately bycatch is recorded and/or reported.

The issue of fleet-wide versus vessel-specific fee structures is especially critical. Significant variation in bycatch rates among vessels exists within many fishing fleets [38], [39] suggesting that taxing an entire fleet uniformly would reduce individual incentives to lower bycatch and be unfair to captains currently minimizing their bycatch. Tax incentives to limit bycatch may also act as incentives to under-report bycatch. Imposing fees based on bycatch by each vessel or the entire fleet would require assurance that bycatch is recorded and/or reported accurately. On-board observer programs, the traditional means of bycatch monitoring, are expensive to operate and involve considerable uncertainty, as bycatch rates from a subset of vessels (typically <5% of a fleet) are extrapolated to estimate bycatch rates for an entire fleet [2], [40]. Thus, CMMB would need to account for the cost of substantial observer coverage – perhaps every boat in a fleet – in order to levy bycatch fees on a vessel-specific basis and thereby maintain individual incentives to reduce bycatch. CMMB may also only be economically viable for a small subset of fishing operations, e.g., industrial fleets targeting lucrative tuna or toothfish that are able to pay the bycatch tax plus associated costs of observers. The tax for bycatch approach may not be a viable option for less lucrative fishing operations, e.g., smaller scale fleets targeting less valuable species.

### 5) CMMB for species meeting criteria 1 through 4 should not increase bycatch impacts to other at-risk species without adequate compensation

As presented by W&D, CMMB is a single-species approach to compensate for (or offset) the impacts of bycatch. However, bycatch is virtually always a multi-species problem (but see Wilkinson et al.[41]); a single fishery often captures dozens of non-target species, including many of conservation concern [42]–[44]. For example, 56 species of sharks are unintentionally caught within the Australian northern prawn fishery [43] and the longline ETBF fishery (the focus of W&D's case study) takes not just FFSH, but also an assortment of other threatened and at-risk species. Among the 10 seabird and 3 sea turtle species documented in the bycatch of the ETBF fishery [45], 7 of the bird and all of the turtle species are categorized as ‘threatened’ by the World Conservation Union (IUCN, www.iucnredlist.org), including the endangered yellow-nosed (*Thalassarche chlororhynchos*) and black-browed (*T. melanophrys*) albatrosses, and the critically endangered leatherback (*Dermochelys coriacea*) and hawksbill (*Eretmochelys imbricata*) sea turtles.

The multi-species nature of fisheries bycatch creates challenges for direct, indirect, or compensatory mitigation approaches. Most direct mitigation techniques benefit only a single taxon, and indeed, some can actually increase mortality for non-targeted taxa [46]. There is a fundamental disparity between the potential conservation benefit of a fisheries closure, which eliminates bycatch mortality for all species taken in the fishery, and that of CMMB, which allows continued bycatch mortality for all species while attempting to offset mortality for a limited subset.

In summary, mitigating bycatch for one or a few species while neglecting the impact of fishing practices on many other non-target species is ecologically unsound, and for CMMB to be effective, impacts to other incidentally captured species need to be evaluated and addressed. One of the most encouraging trends in marine conservation has been the cooperation of agencies and managers concerned with different taxonomic groups to craft bycatch control and reduction plans that would benefit multiple impacted species (e.g., Project Global, http://bycatch.env.duke.edu/). A potential risk of the CMMB approach would be to prolong a single species approach, detracting from a more ecologically-meaningful multi-species perspective.

### Re-Analysis of a Case Study

We were perplexed by the striking results of W&D's quantitative case study on FFSH, which showed an exceptionally strong benefit of offsetting adult mortality with increased chick survival [6]. The mismatch between general life history patterns and this published example prompted us to recreate and then elaborate on models used by W&D in order to understand how CMMB (e.g., rat eradication to increase chick survival) could be so beneficial when the life history of these birds would suggest otherwise.

W&D's CMMB case study examined the trade-off between rat eradication on Lord Howe Island and closure of the ETBF to mitigate for mortality of FFSH from longline bycatch [6]. We evaluated W&D's model assumptions and results in great detail, running both the model they describe (Model 1) and a revised model that corrects several faulty biological assumptions and mathematical errors in their published analysis (Model 2, see Supplement S1 for a detailed accounting of our modeling assumptions, procedures, and results). Finally, in light of the paucity of data for the FFSH population, we ran additional models that incorporated uncertainty in demographic parameters (Model 3a) and in demographic parameters and bycatch rates (Model 3b) to obtain more robust estimates of the potential value of decreases in pre-fledging and adult mortality.

To allow direct comparisons with W&D's reported results, in all our models, we used a deterministic six-stage matrix model with a pre-breeding census, as did W&D. Annual survival is modeled for 5 prebreeder stages (*S_i_*) and for adults (*S_A_*), defined as age six or greater (eqn. 1 in Supplement S1). Reproductive output is the probability that a female fledges a female chick and the chick survives until the next breeding season. It is calculated as the product of the following six probabilities: 1) the probability of an adult female breeding (*p_b_*); 2) the probability of an adult female laying an egg (*p_e_*); 3) the probability of a newly-laid egg hatching and the chick surviving to fledge (*p_f_*); 4) the probability of both parents surviving the reproductive period (*S_r_*), which is a requirement for chick survival to fledging; 5) the probability of the fledgling surviving until one year from egg laying (*S_0_*); and 6) the probability that the chick is female (0.5). Bycatch reduces *S_A_* and *S_r_*, while rats are alleged to depress *p_f_*. W&D assessed the influence on the asymptotic annual population growth rate (*λ*) of eradicating or controlling rats versus reducing bycatch through partial to full fishing area closures. For our comparison, we examined four conditions: status quo (i.e., current bycatch and rat predation effects), eliminating rats, eliminating bycatch, or eliminating both rats and bycatch.

As noted above, for our Model 1 results, we used exactly the model structure and parameter values that W&D detail in their publication [6]. In Model 2, we correct several mathematical and biological flaws we detected in W&D's model. For example, in estimating *S_0_*, W&D erroneously considered the 7-month period from fledging until the end of the next annual census to be 2 months. They also applied an estimate of the total prebreeder survival of 0.32 to the period from age 1 through age 5, when it appears to describe the period from fledging through age 5 (FFSH fledge at 5 months of age). And, although W&D appropriately define *S_r_* as the probability that both parents survive the breeding season, they estimate *S_r_* as the probability that just one parent survives. In addition, according to W&D's apparent source for bycatch rates [13], bycatch data were tallied for September-May, and thus only a portion of this 9-month bycatch impact would affect *S_r_* during the 5-month breeding season (December–April [14]). See Supplement S1 for details of these and other problems and how we corrected them.

Beyond these analytical problems, W&D's analysis of CMMB for FFSH on Lord Howe Island critically depends on the dubious assumption that rat predation substantially depresses FFSH reproductive success and that eradicating rats would eliminate *all* egg and chick mortality except that caused by death of a parent (i.e., *p_f_* = 1.0). While rats are known to prey heavily on FFSH congeners on other islands [47], W&D's primary source for reproductive data on FFSH on Lord Howe Island, Priddell et al. [14], dismisses rat impacts as unsubstantiated and insignificant, and instead focuses on loss of nesting habitat due to increased urbanization as a likely source of declining numbers of breeding pairs. Indeed, Priddell et al. [14] state: “productivity…[was] not suggestive of a population suffering a high rate of predation, and there was no direct evidence of rats preying on flesh-footed shearwater eggs or chicks.” The species of rat on Lord Howe Island, the black rat (*Rattus rattus*), typically affects only small burrow-nesting seabirds (<260 g) [48], and the larger FFSH (580–750 g) may be too large to be highly vulnerable. Also, the FFSH breeding sites on Lord Howe Island are located in areas where rats are already intensively controlled (to protect residences and the local palm industry) (D. Priddel, pers. obs.). Furthermore, the observed breeding success of FFSH on Lord Howe Island (0.51) [14] is within the range reported for other shearwater populations with no known terrestrial predation by invasive species (Table 2 in Supplement S1). Thus, attributing *all* egg and chick mortality observed in this population to rat predation is highly questionable. Assuming that rat eradication would boost *p_f_* to 1.0 is of even greater concern. In our review of the literature, we can find no reports of long-term mean *p_f_* that even approach 1.0 for shearwater colonies, regardless of the presence of rats or other predators (Table 2 in Supplement S1). Consequently, in our Model 2 we use the average breeding success of sooty shearwaters (*P. griseus*) from predator-free Tuhawaiki Island [49] and references therein] of 0.63 as our estimate of *p_f_S_r_*. Assuming these birds experience the same high bycatch mortality as FFSH, we estimate *p_f_* in the absence of rats as 0.748 for Model 2 (Table 1 in Supplement S1; see Supplement S1 text for detailed explanation of estimation procedures).

The results from Models 1 and 2 are simple λ values from deterministic matrices and are based on the implicit assumption that all parameter values used are correct. In Model 3 we investigate the robustness of predictions to uncertainty in parameter values. For each of several different scenarios with different assumptions regarding management and FFSH biology, we generated 10,000 matrices by randomly selecting each demographic rate from a uniform distribution bounded by estimated lower and upper endpoints, and assuming no correlation between rates (see [50]–[52] for similar approaches to the exploration of model uncertainty with limited data). For survival rate estimates, we rely on the parameter ranges given for FFSH by Baker and Wise [13], the same source used by W&D for mean survival rate estimates. For fledging probability, we use bounds based on W&D's assumptions or our more realistic assumptions as respective endpoints (Table 1 in Supplement S1). Because of the difficulty in estimating a range of reasonable bycatch rates, we ran Model 3 simulations both without (Model 3a) and with (Model 3b) uncertainty in bycatch rates. Fisheries-related FFSH mortality appears to have declined from 2001–2005 [53], and thus we considered the bycatch rates assumed by W&D as an upper limit for runs incorporating uncertainty (Table 1 in Supplement S1).

**Figure 5:**
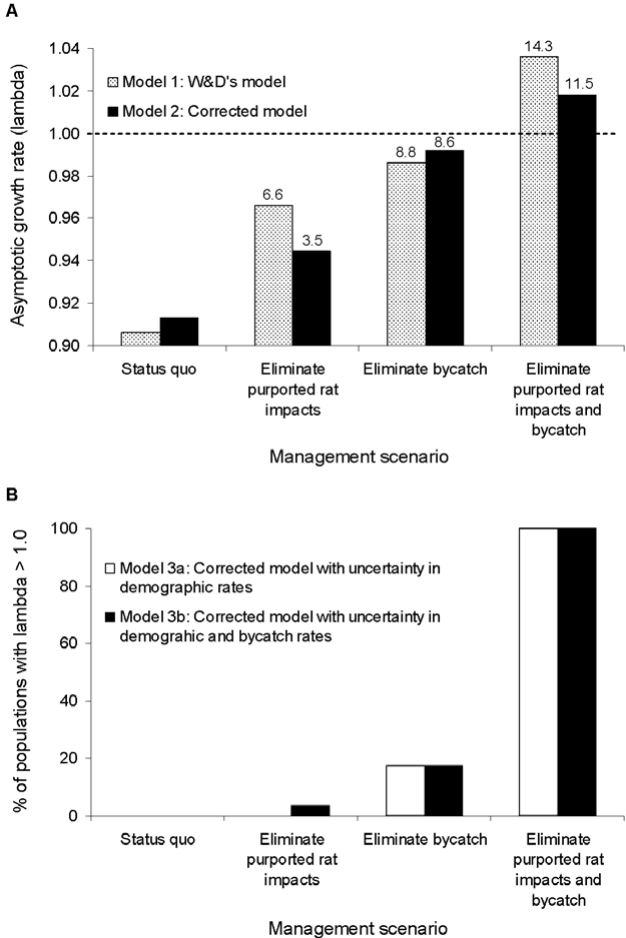
Bycatch elimination consistently yielded greater increases in flesh-footed shearwater (*Puffinus carneipes)* population viability than reducing pre-fledging mortality. A) Predicted asymptotic growth rate (λ) for flesh-footed shearwaters on Lord Howe Island under four management scenarios, using either Model 1, the model described by W&D [6] or Model 2, our corrected model. Values above bars give percentage increase in λ relative to the status quo. Dotted line is λ = 1 (a stable population). B) The percentage of 10,000 replicate matrices for which predicted λ exceeds 1.0 using our corrected model with the inclusion of uncertainty only in demographic rates (Model 3a) or in both demographic and bycatch rates (Model 3b). A λ>1.0 is necessary for a recovering population (see Supplement S1 for detailed description of modeling assumptions, procedures, and results).

We were unable to replicate either the quantitative or qualitative results W&D report using Model 1 (Figure 5A), which employs exactly the same assumptions, model structure, and parameter values they described [6] (Supplement S1). W&D's model (Model 1), along with our corrected Model 2 both predict that in the absence of bycatch reduction, conservation targeting FFSH reproductive success can never boost λ above 1.0, the level necessary for population increase. In addition, increasing adult survivorship via bycatch reduction consistently yielded greater increases in λ than reducing pre-fledging mortality for all models considered (Figure 5A). Model 3, which incorporates the real uncertainty in parameter values, indicates that eliminating bycatch is much more likely to yield a recovering population than enhancing breeding success through terrestrial mitigation (Figure 5B). Importantly, while some Model 3b outcomes indicate that rat eradication does have a very limited potential to bring about population increases, the matrices which yield these results all include substantially lower than average bycatch rates; thus reduced bycatch is ultimately responsible for even these small gains (Figure 5B). In short, the modeling results reported by W&D and used to establish their case for CMMB's efficacy appear to rest upon misinterpretation or misreporting of their results and/or simple programming errors.

Overall, W&D assume exceptionally high impacts of rats on a population that shows no direct or indirect evidence of rat effects while discounting other known factors contributing to mortality. Consequently, rat eradication on Lord Howe Island is unlikely to substantially enhance FFSH breeding success. Reducing documented terrestrial threats such as habitat loss and degradation appears to be the best strategy for increasing FFSH reproductive output [14]. Moreover, even though improved breeding success on Lord Howe Island may slow the rate of FFSH population declines, our models clearly indicate that management strategies must include bycatch reduction if they are to achieve population growth rather than a continued spiral toward local extinction.

## Discussion

### When could CMMB be effective?

Our careful analysis of CMMB proposals underscores the importance of providing a clear and thorough assessment to show a potential conservation strategy's effectiveness before promoting its use. Our analyses suggest that meeting the basic criteria needed for CMMB to be effective and avoid unacceptable costs to conservation will be exceedingly difficult. As we show, most species seriously impacted by bycatch mortality have life histories and population structures that make the offsetting of adult mortality through improvements in other demographic rates extremely challenging. In addition, mitigatable actions for the great majority of impacted species (sharks and cetaceans) are unknown. Finally, CMMB's single species perspective is perhaps its most important limiting factor for overall conservation efficiency. Taken together, these concerns cast doubt on the potential of CMMB to alleviate the threat of extinction for most marine species affected by fisheries bycatch.

The multi-species nature of most fisheries bycatch is a difficult, yet important, problem. By analogy, one could imagine an argument that the eggshell thinning effects from DDT in bald eagles could have been mitigated by more rigorous prosecution to reduce illegal shootings, rather than the economically expensive banning of DDT. This approach *might* have worked to save the bald eagle (in part because, unlike with CMMB, mitigation would have targeted the most important life history stage), but the continued use of DDT would likely have resulted in the extinction of other bird species (e.g., the brown pelican [54], and peregrine falcon [55]). In this case and with bycatch mortality, effective conservation can only be pursued by recognition of the multi-species nature of the problem.

While we have emphasized what we see as severe limitations of CMMB, a consideration of the types of circumstances and/or species for which this approach could work is worthwhile. One situation in which CMMB could be effective is for cases where bycatch mortality in a particular locality is having disproportionately large effects on an entire population. For example, endangered Pacific loggerhead sea turtles are killed in high numbers (relative to total population size) as bycatch in industrial longline fisheries [4]. Through enforcement of the U.S. Endangered Species Act, the United States-based longline swordfish fishery has implemented mandatory bycatch reduction measures that have proven relatively effective [56], including a seasonal fishery closure once the interaction threshold for loggerheads is exceeded. At the same time, however, recent evidence has demonstrated that Pacific loggerheads also suffer high mortality in a small-scale fishery in a localized area, Bahia Magdalena, on the Pacific coast of Baja California Sur, Mexico [57], [58].

Using a CMMB approach, the United Sates longline swordfish fishery could be permitted to continue fishing after exceeding the current maximum allowable loggerhead interactions if fees levied for loggerhead impacts were used to reduce loggerhead bycatch in the small-scale Mexican fishery, possibly including payments to forgo fishing in Bahia Magdalena altogether. If this overall reduction in bycatch resulted in a positive growth rate for the endangered Pacific loggerhead, a CMMB approach could potentially meet criteria 1 through 4. Careful assessment of the multi-species impact from both fisheries – the United States longline swordfish and Mexican small-scale – would have to be conducted to assure that compensatory mitigation measures did not result in a net increase of bycatch for any at-risk species in order to satisfy criteria 5 (e.g., CMMB approaches must not imperil other at-risk species). Most seriously, a CMMB strategy for loggerheads should not result in increased bycatch of the critically endangered Pacific leatherback sea turtle, predicted to go extinct within this century due to high bycatch mortality [59]. Prior to CMMB implementation, a thorough and detailed demographic analysis would need to be conducted to confirm a high likelihood of success for loggerheads under this strategy, and to assess the balance of impacts on other declining species (e.g., leatherback sea turtles).

A second situation where CMMB might merit consideration is as a means of protecting endangered salmon populations. In a less traditional definition of bycatch, individuals of endangered runs are killed ‘incidentally’ by salmon fishing vessels since targeting salmon from only non-endangered runs is impossible. Because salmon runs have been shown to be more threatened by degradation of riparian (i.e., terrestrial) habitats than by harvesting, restoration activities such as the purchase of water rights to ensure minimal stream flows or carefully designed hatchery programs, may be able to compensate for substantial adult mortality [60]. Salmon populations would likely respond to this management strategy in part because they are highly-fecund and short-lived. While these conditions could be ripe for a CMMB-like tradeoff analysis, they also highlight the differences between the life history characteristics of salmon and the species typically impacted by high seas bycatch.

### Conclusions

Our focus here has been on the biological criteria that a CMMB program should meet in order to be seriously considered as a conservation strategy. However, it is also worth returning to the original rationale for CMMB, which is largely an economic one. Essentially, this argument is that the economic expense of direct bycatch reduction is too onerous to be seriously pursued, and thus we need less direct, less expensive methods to conserve the multiple species that are rapidly declining due to high bycatch mortality. While the economic costs of reducing bycatch are substantial, many successful conservation mandates enacted over the past 150 years directly reduced mortality of at-risk species and often had very large economic costs. Among these were The Migratory Bird Treaty Act of 1918, International Convention for the Regulation of Whaling (1946), and the international ban on ivory through the Convention on International Trade in Endangered Species of Wild Fauna and Flora (CITES) (1989). As these past successes show, successful conservation actions can be pursued even when they have substantial economic costs. Thus, in cases where alternative strategies have little likelihood of success, we should not be averse to promoting conservation strategies that impose short-term economic costs but that will actually work.

Although our review reaches a pessimistic conclusion about the effectiveness of trading bycatch mortality for terrestrial mitigation activities, we do not disagree that a united analysis of different conservation measures for bycatch-impacted species is needed. Tackling conservation threats both at sea and on breeding sites has long been advocated to promote recovery of threatened and endangered marine species [61], [62]. Such parallel efforts benefit island as well as marine ecosystems, where many rare and endemic species are threatened with extinction [63]. We strongly support funding for island restoration as part of a comprehensive approach to conservation and expect that in many situations both bycatch reduction and non-native species removal will be necessary to conserve some terrestrially breeding marine species. However, exchanging firm bycatch limits for local eradication of exotic nest predators is likely to harm a broad suite of vulnerable marine species while offering only marginal benefits for species breeding on islands. In summary, although we readily acknowledge that a multi-strategy approach to ameliorate the effects of bycatch mortality is needed, we do not believe the “avoid, minimize, and offset” hierarchy of approaches set forth in the Convention on Biological Diversity and proposed by Donlan and Wilcox [15] trumps the fundamental goal of reducing fisheries bycatch to ensure the persistence of vulnerable marine taxa.

**Figure 6:**
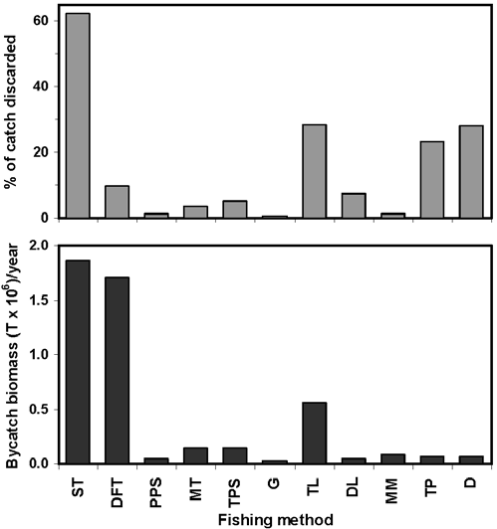
Percentage of harvest discarded as bycatch (mammals, birds, turtles, sharks, bony fish and invertebrates) for each major marine fishing method. Fishing methods are abbreviated as follows: ST = shrimp trawl; DFT = demersal finfish trawl; PPS = small pelagic purse seine; MT = midwater trawl; TPS = tuna purse seine; G = gillnet; TL = tuna and highly migratory species longline; DL = demersal longline; MM = multispecies or multigear; TP = mobile trap or pot; D = dredge. Data for handlines, tuna pole and line, hand collection, and squid jig methods are not shown, but their percentage of catch discarded is ≤2% of the annual bycatch biomass. Data from Kelleher [68].

The global problem of fisheries bycatch requires innovative, yet carefully vetted, conservation approaches. Until a detailed plan of how CMMB would be implemented has been released – including details of fee structure, bycatch recording, reporting, and estimation – consideration of this type of approach is premature. The fishing industry removes vast amounts of biomass as bycatch from the ocean each year (Figure 6) and impacts a multitude of long-lived predators (e.g., sharks, marine mammals, seabirds) critical to the healthy functioning of marine ecosystems [64], [65]. Conservationists, managers, and industry partners should work together to assure that bycatch reduction measures meet a minimum list of criteria to assure that any adopted bycatch reduction strategy results in a positive effect on marine species and communities.

## Supporting Information

Supplement S1 Re-evaluation of the W&D model for flesh-footed shearwaters on Lord Howe Island
(0.55 MB DOC)
