# Supplementary material for: Evaluating the Potential Effectiveness of Compensatory Mitigation Strategies for Marine Bycatch
Source: PLoS One. 2008 Jun 18;3(6):e2480. doi: 10.1371/journal.pone.0002480 (PMC2423618; doi:10.1371/journal.pone.0002480)
Supplement: Supplement S1 — Re-evaluation of the W&D model for flesh-footed shearwaters on Lord Howe Island (0.55 MB DOC) [file pone.0002480.s001.doc]

**Supplement S1: Re-evaluation of the W&D model for flesh-footed shearwaters on Lord Howe Island**

**INTRODUCTION.**

Here we describe and critique the model used by Wilcox and Donlan [1] (henceforth W&D) to compare the demographic gains for flesh-footed shearwaters (FFSH, Figure 1) on Lord Howe Island (LHI) under different management scenarios.

**
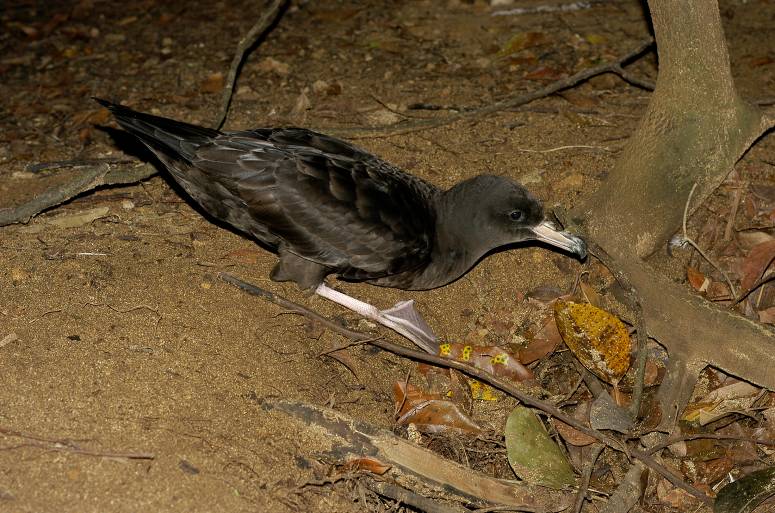
**

**Figure 1. A flesh-footed shearwater entering a burrow on Lord Howe Island.**

**Photo courtesy of Barry Baker.**

Specifically, we compare the benefits to FFSH of eliminating anthropogenic on-island impacts to reproduction (attributed to rat predation by W&D) and eliminating bycatch mortality. Because of numerous, non-trivial problems with both the model structure and the reporting of model results by W&D, we constructed and analyzed three sets of demographic models for this population. Our approach was to first build a model exactly as described by W&D (Model 1). Next, we build a demographic model that remedies several faulty assumptions in Model 1 to provide a more accurate assessment of the effects of alternative management options (Model 2). Finally, we run models that include parameter uncertainty to more thoroughly explore the range of likely outcomes given the uncertainties surrounding estimates of both demographic parameters and management effectiveness (Model 3). As has been repeatedly emphasized in the population viability analysis and wildlife management literatures [2-5], inclusion and analysis of uncertainty is required to assess management effects in the face of substantial data gaps like those in the FFSH case study.

While many additional complexities might improve the realism and reduce the bias of the deterministic, density-independent models that W&D used, we consider the basic approach adequate for a general analysis of the relative merits of eliminating terrestrial anthropogenic impacts vs. bycatch. In our work here, rather than estimating the relationship between management effort, cost, and change in asymptotic population growth rate (λ), we focus on a comparison of predicted λ values under four scenarios: status quo, eliminating terrestrial anthropogenic impacts, eliminating bycatch, and eliminating both terrestrial impacts and bycatch. All but the last were analyzed by W&D and formed the basis of the results they reported.

**MODEL STRUCTURES.**

W&D build a deterministic, six-stage matrix model, with reproduction commencing when individuals turn six, apparently based on the model of Keitt et al. [6] for black-vented shearwaters (*Puffinus opisthomelas*) on Natividad Island, Mexico. Although the only previous modeling of FFSH [7] assumed breeding started at age seven, the timing of the first reproductive attempt is variable within seabird populations, and thus, to help simplify comparisons between model outputs, we use the following matrix, identical to that of W&D:

eqn. 1

where *S_i_* values are annual survival rates from age *i* to *i+1*, *S_A_* is annual adult survival rate, and *F_A_* represents net annual reproductive output, as described next. Although we used this fixed age at first reproduction in all our models, we note that the variability surrounding breeding age and reproductive success in other shearwater populations [8] suggests that the population dynamics of FFSH may be more realistically modeled using a probability of breeding over a broad range of ages (e.g., 2-15 used for short-tailed shearwaters STSH, *P. tenuirostris* by Hunter et al. [9]). Like W&D, our models focus on females only.

W&D break down reproductive output, the probability that a female fledges a female chick and the chick survives until the next breeding season, as follows:

*F_A_ = 0.5 p_b_ p_e_ p_f_ S_r_ S_0_* eqn. 2

*F_A_* is calculated as the product of the probability of breeding for birds age six and greater (*p_b_*), the probability of laying an egg (*p_e_*), the probability of a newly-laid egg hatching and the chick surviving to fledge given that both parents survive (*p_f_*), the probability of both parents surviving the reproductive period (without two parents a chick is nearly certain to die, *S_r_*), the probability of the fledgling surviving until one year from egg laying (*S_0_*), and the probability that the chick is female, or 0.5. We use this same equation to estimate *F_A_* in all models, but correct various errors with the estimation of the values or functions that define these probabilities in Models 2 and 3. W&D use a pre-breeding census model, and because they assume that all breeding females lay an egg, their model implicitly assumes the year starts immediately prior to egg laying. We use the same approach.

**SURVIVAL RATES.**

Next, we describe the data and assumptions behind W&D’s parameter values, as well as the flaws in these estimates that we sought to correct in Models 2 and 3. While some of these problems are trivial, others are biologically unrealistic and strongly affect the results of the models. Thus, we proceed through their methods with careful explanations of their and our assumptions and data sources.

Prebreeder survival. W&D estimate annual prebreeder survival, *S_1-5_*, as 0.80, based on the multi-year prebreeder survival estimate, *S_pb_*, of 0.32, which they assume applies to the period from *S_1_* to *S_5_* (i.e., 0.32^1/5^=0.80, Table 1). Fledgling survival, *S_0_*, was estimated by W&D as based on the same annual rate as older prebreeders (i.e., 0.80), but “adjusted to cover the period from fledging (age 10 months) to first birthday”, thus 0.80^2/12^=0.96). We note two problems with W&D’s estimate of *S_0_*. First, FFSHs fledge at five months, thus, using their logic of adjusting the annual prebreeder survival rate, *S_0_* should be 0.80^7/12^, or 0.895. Second, W&D’s stated source for their prebreeder survival rate [7], give a lower estimate of *S_0_* as 0.50. While the source of this lower *S_0_* is unclear, we note that the multi-year prebreeder survival estimate of 0.32 appears to apply to the period from fledging until breeding [9,10]. Thus, for Model 2, we estimate annual prebreeder survival rates based on an overall transition rate for the 67 months from fledging to age six of 0.32, or 0.32^12/67^=0.815 for *S_1-5_* and 0.32^7/67^=0.888 for *S_0_* (Table 1). For Model 3, lower and upper endpoints for *S_0_* and *S_1-5_* are based on endpoints for *S_pb_* of 0.30 and 0.34 [7].

Adult survival. Adult survival was estimated as the product of the probability of an adult surviving all mortality sources other than bycatch, *S_Ad_*, and the probability of an adult surviving bycatch mortality, *S_By_*. Like W&D, we used an estimate of 0.94 for *S_Ad_*; this estimate, obtained from Baker and Wise [7], appears relatively high based on published survival rates for shearwaters [11]. Nonetheless, a high survival rate is warranted given that it characterizes conditions in the absence of fishing-related mortality. For Model 3, we use the parameter ranges given for FFSH by Baker and Wise [7] (Table 1).

Bycatch effects. In W&D’s models, bycatch mortality only afflicts adults, following Baker and Wise [7]. We use the same approach, but note that bycatch of juveniles is likely to be another source of mortality for this population. W&D estimated the relationship between area closed to fishing and bycatch mortality by fitting a Poisson regression model including year, month, and timing of fishing operations (day vs. night) to bycatch data for 2001 through 2005. They then fixed year to 2002, month to November, and fishing to day to estimate the reduction in bycatch with area closure under these conditions, which correspond to high bycatch rates. We examined only scenarios of fully open or completely closed fisheries, and we sought to use bycatch rates as similar to those of W&D as possible for these endpoints. W&D do not give an estimate of *S_By_*, and they provide conflicting numbers of adults killed with open fishing, stating 4500 killed in the absence of management but also using the observed 2002 bycatch of 4783 birds killed for estimating the effects of fishing on reproduction via parental mortality (see descriptions of *S_r_* below). To maintain internal model consistency with this *S_r_* value, we assumed 4783 FFSH were killed in open fishing. Thus, taking W&D’s assumption of 17462 breeding pairs in 2002 and adjusting for *p_b_*, we obtain a total adult population size of 46565 and use this to estimate *S_By_*, the probability of surviving bycatch mortality with open fishing, as 0.897. We assume that W&D used a similar value. We optimistically assume that closure of the fishery eliminates all shearwater bycatch, consistent with W&D, although we note that FFSH are taken in other fisheries outside of the breeding season.

Because of the difficulty in estimating a range of reasonable bycatch rates, we ran Model 3 simulations both with and without uncertainty in bycatch. W&D give a range of 1800-4500 FFSH killed annually from 1998 through 2002 [7,12]. However, this range does not represent low and high extremes over these years, but is instead the estimated mean low and high annual FFSH bycatch assuming either all nighttime (1800) or all daytime (4500) fishing. The mean number of FFSH killed annually, based on the actual distribution of day and night fishing effort over this time period, was 3700 [7]. Oddly, W&D do not provide a bycatch range based on the 2001 to 2005 data that they actually analyzed, although fisheries-related FFSH mortality appears to have declined substantially during this time period, with estimates of as few as 31 birds killed in 2004-2005 [13]. Clearly, such minimal bycatch impacts would negate the need for considering fishery closures at all. Although these low estimates are likely biased by observer coverage [13,14], there nonetheless appears to be substantial variation in bycatch rates over time, and mean annual bycatch is likely to be lower than the value of 4783 assumed by both W&D and ourselves. Thus we considered models in which annual bycatch ranged from 1800 to 4783 (*S_By_*=0.897-0.961) to represent a range of mean rates expected under different fleet-based mitigation measures.

**REPRODUCTIVE RATES.**

We used W&D’s estimates for the probability that FFSH breed and lay eggs (*p_b_* and *p_e_*, Table 1). Shearwaters lay one egg per clutch [15] and we were unable to locate enough data to justify including uncertainty around the estimate of probability of breeding for our Model 3 simulations. Estimating the components of breeding success with and without management actions are the most complex for these analyses, and are also where W&D make the most dubious assumptions regarding the biology of shearwaters. Thus, we first present the calculations behind W&D’s parameter values, and then explain the problems with their assumptions.

W&D’s breeding success (egg to fledge survivorship) estimates. W&D take advantage of detailed burrow surveys conducted by Priddel et al. [12] at the egg stage and 96 days later at the late chick stage to estimate a rate for egg to fledge survivorship. W&D appropriately adjust the eggs per burrow estimate of Priddel et al. [12] to account for eggs missed by observers during the egg survey but assumed present due to the discovery of a chick at the late chick survey. They then calculated the number of fledges per egg by dividing fledges per burrow by eggs per burrow to yield an observed fledges per egg of 0.476. Finally, they extrapolate this value to a 145-day period to account for the 28 days from the peak of egg laying until the egg survey and the 21 days from the late chick survey until estimated fledging. Thus:

*p_f_S_r_* = (0.476^145/96^) = 0.326 eqn. 3

Although W&D define *S_r_* as the probability that both parents survive the breeding season, a requirement for successful incubation and chick rearing, in their equation (eqn. 4) they estimate *S_r_* as the probability that one parent survives. They make this estimate by multiplying the 2002 bycatch survival rate, *S_By_*, of 0.897 by the adult survival rate, *S_Ad_*, of 0.94, adjusted for the 5 month breeding season, or:

*S_r_* = *S_By_S_Ad_*^5/12^ = 0.8970.94^5/12^ = 0.875 eqn. 4

Factoring *S_r_* out of eqn A3 yields an estimate of *p_f_* of 0.373, which W&D state incorrectly as 0.378. Finally, W&D assume that in the absence of rat predation, *p_f_* is 1.0. While the only parameter estimate W&D provide for *S_r_* is 0.875, we assume that with fishery closure they adjust this to exclude the effects of bycatch mortality, leaving *S_r_*=0.94^5/12^=0.975.

Corrections to W&D’s breeding success estimates. We question several of the assumptions behind the calculations W&D used to estimate breeding success for FFSH on LHI under different management scenarios. First and foremost, the assumption that *all* direct mortality of eggs and chicks is attributable to rat predation is deeply flawed, being at odds with shearwater biology in general and detailed studies of the FFSH Lord Howe population in particular. While rats are known to prey heavily on some seabird colonies [16], W&D’s primary source for reproductive data on FFSH on LHI dismisses rat impacts as unsubstantiated and insignificant, and instead focused on loss of nesting habitat due to increased urbanization as a likely source of declining numbers of breeding pairs [12]. Indeed, Priddel et al. [12] state: “productivity…[was] not suggestive of a population suffering a high rate of predation, and there was no direct evidence of rats preying on flesh-footed shearwater eggs or chicks.” Furthermore, the breeding success of FFSH on LHI is within the range of values reported for other shearwater populations not experiencing terrestrial predation by invasive species (Table 2). Thus, W&D assume exceptionally high impacts of rats on a population that shows no direct or indirect evidence of rat effects while discounting other known factors contributing to mortality.

Before constructing better estimates of survival to fledging, we must deal with some less critical aspects of parameter estimation. W&D divide fledges per burrow by eggs per burrow to get fledges per egg, but the former excludes burrows observers failed to relocate at chick surveys, while the latter, based on our reanalysis, appears to include eggs from such burrows; their miscalculation slightly depresses the overall fledges per egg, which they estimated at 0.476 and we estimate as 0.501. In addition, because of the great variation in colony size across LHI (53-7779 breeding pairs per colony), we felt a more appropriate approach was to estimate fledges per egg as a weighted average based on colony-specific rates and the number of breeding pairs per colony, yielding an observed fledges per egg of 0.513.

As explained above, W&D adjust observed fledges per egg to account for early and late season mortality. We consider this extrapolation, which assumes a constant daily mortality rate for all ages of eggs and chicks, to be unrealistic, since the mortality rate for shearwater eggs and hatchlings is known to vary throughout the breeding season. In particular, older hatchlings, estimated to be two to four weeks from fledging at the final chick survey, are expected to have relatively high survival rates compared to eggs and young chicks [10,15,17]. In addition, many or most studies of breeding success are based on a final survey at the late chick stage, and these are seamlessly paired with estimates of prebreeder survival from this late chick stage until return to breeding. Indeed, the estimated *S_pb_* of 0.32 used in this study is a measure of survival from late chick surveys until breeding [10]. Even though we disagree with W&D’s extrapolation of Priddel et al.’s mortality estimates to the entire breeding season and the inherent assumption of constant daily mortality rates from egg to fledge, for comparison of model results, we include this bias in estimation of *p_f_S_r_* and account for the missed 28 days before egg sampling and the 21 days after late chick sampling following the method of W&D, yielding a *p_f_S_r_*=0.513^145/96^=0.365. We use this value in Model 2. However, for our Model 3, which incorporates parameter uncertainty, we set the upper range of *p_f_S_r_* at 0.423, a value that omits the late season mortality extrapolation (i.e., 0.513^124/96^).

According to Baker and Wise [7], the apparent source for W&D’s bycatch rates, bycatch data are tallied for the Austral summer of September-May, and thus only a portion of the bycatch impact would affect reproduction. Assuming constant rates of bycatch across these 9 months, we adjust *S_r_* to the 5-month breeding season (December-April) [12]. Unlike W&D, we estimate *S_r_* as the probability that both parents survive the breeding season, or the product of the survival probabilities for each parent, which we assume to be equal. Consequently, we find:

*S_r_* = (*S_By_*^5/9^*S_Ad_*^5/12^)^2^ = (0.897^5/9^0.94^5/12^)^2^ = 0.842 eqn. 5

Solving *p_f_S_r_* for *p_f_* yields *p_f_* =0.434 if *p_f_S_r_* is extrapolated to account for both early egg and late chick survival (Model 2, lower bound in Model 3) or 0.502 if late chick extrapolations are excluded (upper bound in Model 3).

While W&D use a clever approach to the problem of factoring out the influence of parental mortality on chick survival and fledging success, fledging probability is known to be highly variable in shearwaters, with annual *p_f_S_r_* values ranging from 0.25 to 0.83 for one STSH colony [10], making estimates of mean rates based on a single year’s data [12] problematic. These estimates are slightly lower than those estimated for FFSH on Woody Island in Western Australia, which like LHI, has rats but no evidence of predation on shearwaters. Based on daily burrow checks through the entire breeding season, this population had a mean *p_f_S_r_* for 2001 and 2002 of 0.468 [18], suggesting that the values we and W&D use for *p_f_* in the presence of rats may be conservative.

Finally, we return to the assumption that most biases W&D’s results – that in the absence of rat predation egg and chick survival (*p_f_*) is 1.0. We can find no reports of long-term mean *p_f_* that even approach 1.0 for shearwater colonies, regardless of the presence of rats or other predators. With a *p_f_* of 1.0, breeding success would be 0.842 if bycatch occurred at rates comparable to those of FFSH on LHI and 0.950 for a population not affected by bycatch (compare to breeding success rates in Table 2). While W&D report an upper value of 0.93 for productivity [19], they do not provide a source for it; the only comparable value we located was a one-year breeding success of 0.93 reported for 15 wedge-tailed shearwaters (*P. pacificus*) using nest boxes [20]. Thus, we use the average breeding success of sooty shearwaters (SOSH, *P. griseus*) from predator-free Tuhawaiki Island [11 and references therein] of 0.63 (Table 2) as our estimate of *p_f_S_r_* in Models 2 and 3. This is appropriate due to the colony’s proximity to LHI and the extensive reliance of our models on parameter estimates derived from data on SOSH (Table 1). Assuming these birds experience the same high bycatch mortality as FFSH, we estimate *p_f_* in the absence of rats as 0.748 for Model 2, and set this as the lower bound of *p_f_* in Model 3 simulations. The highest reported *p_f_S_r_* we found in the literature for related species was 0.70 for Manx shearwaters in Wales, UK [21], which corresponds to a *p_f_* of 0.831 if bycatch rates are similar. Although the ecological setting differs substantially, we use this as the upper endpoint in our analyses incorporating uncertainty (Model 3).

**GENERATION OF MODEL OUTPUTS.**

Model 1 and 2 results are simple λ values from deterministic matrices. To investigate the robustness of predictions to uncertainty in parameters in Model 3, we generated 10,000 matrices by randomly selecting from a uniform distribution bounded by the estimated lower and upper endpoints for mean demographic rates, assuming no correlation between rates (see [4,9,22] for similar approaches to the exploration of model uncertainty with limited data). As described above, for survival rate estimates, we rely on the parameter ranges given for FFSH by Baker and Wise [7], the same source used by W&D for mean survival rates estimates. For fledging probability, we use the values based on W&D’s assumptions or our more realistic assumptions as respective endpoints (see text, Table 1).

**RESULTS.**

Model 1, constructed exactly as described by W&D, predicts a population declining rapidly, with a λ of 0.906 (Table 3). Eliminating terrestrial anthropogenic impacts increases λ by 6.6%, but eliminating bycatch results in greater gains in λ of 8.8%. Our Model 2, which corrects the most serious flaws in W&D’s approach, predicts a λ of 0.913 for current conditions, with elimination of terrestrial impacts raising λ by only 3.5% but elimination of bycatch boosting it by 8.6% to 0.992, or a nearly stable population. Only elimination of both bycatch and terrestrial anthropogenic impacts yields a growing population, with a λ of 1.018.

W&D report that eliminating terrestrial impacts (their rat eradication) and bycatch increase λ by 32% and 6% respectively, but after 20 years the relative efficacies of these management strategies are nearly equal, with λ increasing by 64% and 63% respectively. Using their reported model (our Model 1), we are unable to replicate anything close to this assessment, although we note that trajectories of the total female population size through time include a short-term beneficial effect of eliminating terrestrial anthropogenic impacts on total numbers (not λ), with numbers nearly converging under both strategies after 20 years (Figure 2A). This temporary increase in the numbers of females, which arises from the unrealistically high *p_f_*, slowly dissolves as birds become adults and suffer bycatch mortality at sea. We emphasize that when using a more realistic (yet still optimistic) value of *p_f_* in the absence of terrestrial anthropogenic impacts (Model 2), even this temporary gain does not occur (Figure 2B).

**
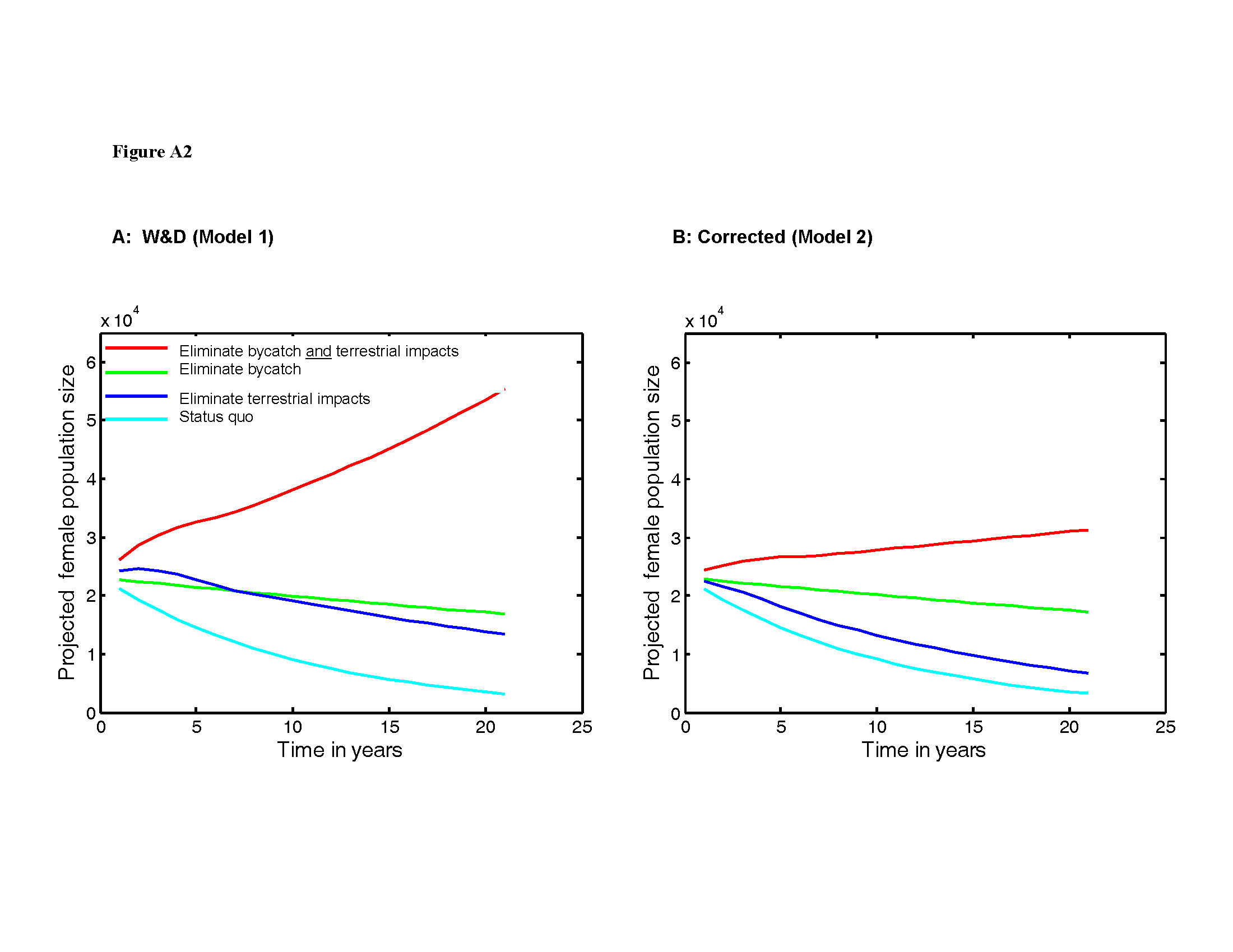
**

**Figure 2. Population trajectories based on deterministic matrix models for flesh footed shearwaters on Lord Howe Island.** Population trajectories represent four scenarios (status quo, eliminating terrestrial anthropogenic impacts, eliminating bycatch, and eliminating both terrestrial impacts and bycatch) based on models described in Wilcox and Donlan [1] (A) and corrected models described in this study (B) (Table 1). Consistent with Wilcox and Donlan [1], populations start at a stable age distribution given 17,462 breeding pairs.

**
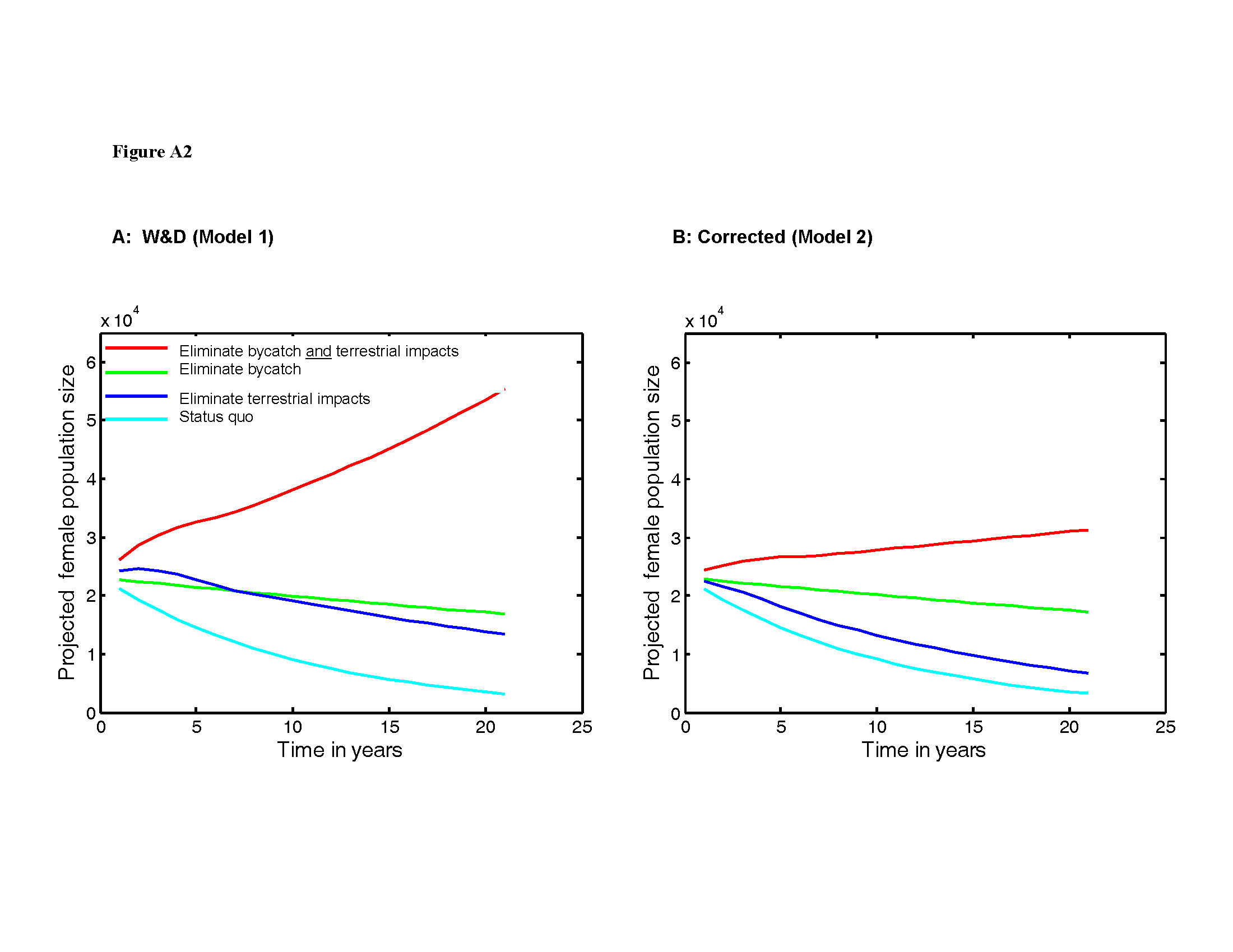
**

W&D’s claims of large percentage increases in annual growth rates when eliminating terrestrial anthropogenic impacts appear to be the result of flawed model structures combined with the use of some measure of population dynamics other than λ. Their reported results are inaccurate both quantitatively and qualitatively, and they create a dramatically incorrect impression of the efficacy of reducing terrestrial impacts for the persistence of FFSH populations.

Using our revised parameter values and incorporating parameter uncertainty in survival rates and probability of fledging (Model 3a, Figure 3, left column), we find that eliminating terrestrial anthropogenic impacts never results in predictions of λ>1. In contrast, bycatch elimination yields λ>1 in 17.4% of matrices. Employing both strategies always yields λ>1. When also including parameter uncertainty in mean bycatch rates (Model 3b, Figure 3, right column), 3.4% of matrices for eliminating terrestrial anthropogenic impacts achieve λ>1 (bycatch elimination performance is unchanged). Importantly, these more optimistic results for eliminating terrestrial anthropogenic impacts occur due to the inclusion of some matrices with lower bycatch rates, and thus reduced bycatch is ultimately responsible for these gains.

Some potentially important complexities excluded from our model are likely to magnify the importance of bycatch reductions. For example, environmental stochasticity, especially weather extremes, influences both fledging probability and the mortality of birds at sea. Because population outcomes for FFSH are particularly sensitive to changes in adult mortality, population resilience in the face of bycatch mortality could be strongly affected by severe weather. In addition, the widowing that occurs when one member of a pair is lost to bycatch has multi-year effects on breeding probabilities [8].

**Model 3a**

**Fixed high bycatch rates**

**Model 3b**

**Uncertainty in bycatch rates**

**Figure 3. Asymptotic growth rates (λ) with parameter uncertainty for deterministic matrices describing demographic rates of flesh footed shearwaters on Lord Howe Island using the corrected models developed in this study (Table 1).** Results are for λ values from 10,000 replicate matrices for four scenarios formed by randomly selecting mean demographic rates from uniform distributions defined by lower and upper values as described in Table 1. Results are shown (a) with and (b) without uncertainty in bycatch rates. Percentages indicate the percent of model runs yielding λ>1.

Our models indicate that improving breeding success by reducing anthropogenic impacts can result in some, nontrivial improvement in λ for FFSH on LHI. However, only bycatch elimination appears to have a reasonable chance of promoting positive population growth. A point to emphasize in all these results is that our models compare bycatch elimination to a poorly defined set of terrestrial impacts – not rat eradication, as W&D attempt to analyze. Because there is no evidence of rat effects on this FFSH population, we analyze the effects of unspecified measures that may be able to raise reproductive success over that currently seen. However, this ambiguity calls into question the validity of compensatory mitigation for marine bycatch (CMMB) as a conservation strategy. Lacking identification of a specific stressor to reproduction, it is impossible to gauge the actual costs or benefits of management actions using the economic framework advocated by W&D. Nonetheless, the costs of broader terrestrial conservation measures such as habitat protection are likely to be far higher than those of rat eradication.

**REFERENCES.**

1. Wilcox C, Donlan CJ (2007) Compensatory mitigation as a solution to fisheries bycatch–biodiversity conservation conflicts. Frontiers in Ecology and the Environment 5: 325-331.

2. Ellner SP, Fieberg J (2003) Using PVA for management despite uncertainty: Effects of habitat, hatcheries, and harvest on salmon. Ecology 84: 1359-1369.

3. Ludwig D (1996) Uncertainty and the assessment of extinction probabilities. Ecological Applications 6: 1067-1076.

4. Wisdom MJ, Mills LS, Doak DF (2000) Life stage simulation analysis: Estimating vital-rate effects on population growth for conservation. Ecology 81: 628-641.

5. Burnham KP, Anderson D (2002) Model selection and multi-model inference. New York: Spring-Verlag. 496 p.

6. Keitt BS, Wilcox C, Tershy BR, Croll DA, Donlan CJ (2002) The effect of feral cats on the population viability of black-vented shearwaters (*Puffinus opisthomelas*) on Natividad Island, Mexico. Animal Conservation 5: 217-223.

7. Baker GB, Wise BS (2005) The impact of pelagic longline fishing on the flesh-footed shearwater *Puffinus carneipes* in Eastern Australia. Biological Conservation 126: 306-316.

8. Bradley JS, Wooller RD, Serventy DL (1990) The influence of mate retention and divorce upon reproductive success in short-tailed shearwaters *Puffinus tenuirostris*. Journal of Animal Ecology 59: 487-496.

9. Hunter CM, Moller H, Fletcher D (2000) Parameter uncertainty and elasticity analyses of a population model: Setting research priorities for shearwaters. Ecological Modelling 134: 299-323.

10. Serventy DL, Curry PJ (1984) Observations on colony size, breeding success, recruitment and inter-colony dispersal in a Tasmanian colony of short-tailed shearwaters (*Puffinus tenuirostris*) over a 30-year period. Emu 84: 71-79.

11. Cuthbert R, Davis LS (2002) Adult survival and productivity of Hutton's shearwaters. Ibis 144: 423-432.

12. Priddel D, Carlile N, Fullagar P, Hutton I, O'Neill L (2006) Decline in the distribution and abundance of flesh-footed shearwaters (*Puffinus carneipes*) on Lord Howe Island, Australia. Biological Conservation 128: 412-424.

13. Stobutzki I, Lawrence E, Bensley N, Norris W (2006) Bycatch mitigation approaches in Australia’s eastern tuna and billfish fishery: Seabirds, turtles, marine mammals, sharks, and non-target fish. Information Paper WCPFC-SC2/EBSWG – IP5 presented at the Ecosystem and Bycatch Specialist Working Group of the Second Meeting of the Scientific Committee of the Western and Central Pacific Fisheries Commission. Manila: Australian Fisheries Management Authority. 34 p.

14. ETBRAG, Eastern Tuna and Billfish Fishery Resource Assessment Group (2005) 17 & 18 November 2005 meeting minutes. Hobart: Australian Fisheries Management Authority. 11 p.

15. Warham J (1990) The petrels: Their ecology and breeding systems. London: Academic Press. 440 p.

16. Jones HP, Tershy BR, Zavaleta ES, Croll DA, Keitt BS, et al. (2008) Severity of the effects of invasive rats on seabirds: A global review. Conservation Biology 22: 16-26.

17. Seto NWH, Conant S (1996) The effects of rat (*Rattus rattus*) predation on the reproductive success of the Bonin petrel (*Pterodroma hypoleuca*) on Midway Atoll. Colonial Waterbirds 19: 171-185.

18. Powell CDL (2004) The breeding biology of the flesh-footed shearwater *Puffinus carneipes*, PhD dissertation. Perth: Murdoch University. 320 p.

19. Wilcox C, Donlan CJ (2007) Reply to: Compensatory mitigation for marine bycatch will do harm, not good. Frontiers in Ecology and the Environment 7: 350-352.

20. Byrd GV, Moriarty DI, Brady BG (1983) Breeding biology of wedge-tailed shearwaters at Kilauea Point, Hawaii. Condor 85: 292-296.

21. Brooke MDL (1978) Some factors affecting laying date, incubation and breeding success of Manx shearwater, *Puffinus puffinus*. Journal of Animal Ecology 47: 477-495.

22. Kesler DC, Haig SM (2007) Conservation biology for suites of species: Demographic modeling for Pacific island kingfishers. Biological Conservation 136: 520-530.

23. Igual JM, Forero MG, Gomez T, Orueta JF, Oro D (2006) Rat control and breeding performance in Cory's shearwater (*Calonectris diomedea*): effects of poisoning effort and habitat features. Animal Conservation 9: 59-65.

24. Pierce RJ (2002) Kiore (*Rattus exulans*) impact on breeding success of Pycroft’s petrels and little shearwaters. Wellington: New Zealand Department of Conservation. 24 p.

25. Thompson KR, Furness RW (1991) The influence of rainfall and nest site quality on the population dynamics of the Manx shearwater *Puffinus puffinus* on Rhum. Journal of Zoology 225: 427-437.

26. Floyd RB, Swanson NM (1983) Wedge-tailed shearwaters on Muttonbird Island: An estimate of the breeding success and the breeding population. Emu 82: 244-250.

27. Harris MP (1969) Food as a factor controlling breeding of *Puffinus lherminieri*. Ibis 111: 139-156.

28. Bourgeois K, Vidal E (2007) Yelkouan shearwater nest-cavity selection and breeding success. Comptes Rendus Biologies 330: 205-214.

29. Keitt BS, Tershy BR, Croll DA (2003) Breeding biology and conservation of the back-vented shearwater *Puffinus opisthomelas*. Ibis 145: 673-680.

**Table 1.** Demographic rates used by Wilcox and Donlan [1] and this study in matrix models for the flesh-footed shearwater (FFSH, *Puffinus carneipes*) on Lord Howe Island. *S_0_* is the survival probability from fledging until one year; *S_1-5_* is the annual survival probability for prebreeders aged 1 through 5; *S_Ad_* is the adult survival probability for birds ≥6; *S****_By_*** is probability adults survive bycatch; *p_b_*is the annual breeding probability; *p_e_* is the probability of laying an egg;*p_f_* is the probability of an egg hatching and the chick surviving to fledging, given that both parents survive, estimated with and without rats present; *S_r_* is he probability of both parents surviving the reproductive period, estimated with and without bycatch mortality occurring. Many parameter estimates are based on studies of similar species, in particular sooty shearwaters (SOSH, *P. griseus*) and short-tailed shearwaters (STSH, *P. tenuirostris*). See text for details on parameter estimates.

| **Parameter** | **W&D [1] value** | **Species** | **This study** | **Range** | **Species** |
| --- | --- | --- | --- | --- | --- |
| ***S_0_*** | 0.960 [7] | STSH, SOSH | 0.888 [7,10] | (0.882 0.893) [7,10] | STSH |
| ***S_1-5_*** | 0.800 [7] | STSH, SOSH | 0.815 [7,10] | (0.806 0.824) [7,10] | STSH |
| ***S_Ad_*** | 0.940 [7] | STSH, SOSH | same | (0.930 0.950)^1^ [7] | SOSH |
| ***S_By_*** | ~0.897^1^ | FFSH | 0.897^1^ | (0.897 0.961)^1^ [7] | FFSH |
| ***p_b_*** | 0.75 [7] | STSH, SOSH | same | -- | STSH, SOSH |
| ***p_e_*** | 1^2^ | FFSH | same | -- | FFSH |
| ***p_f_* rats** | 0.373^1^ [12] | FFSH | 0.434^1^ [12] | (0.434 0.502)^1^ [12] | FFSH |
| ***P_f_* no rats** | 1^2^ | FFSH | 0.748^1^ [11 and references therein] | (0.748 0.831) [11 and references therein] | SOSH on predator free island |
| ***S_r_*bycatch** | 0.875^1^ [7] | FFSH | 0.842 | (0.835 0.917)^1^ [7] | FFSH |
| ***S_r_*no bycatch** | 0.975^1^ [7] | FFSH | 0.950 | (0.941 958)^1^ [7] | FFSH |

^1^ Estimated, see text for methods

^2^ Assumed

**Table 2.** Mean annual breeding success (egg to late chick stage or egg to fledge) values reported for shearwater species based on empirical studies of ≥2 years; data for 1 year on Lord Howe Island included for comparison. Breeding success for colonies in which invasive predators are absent or controlled is given first, followed by values for colonies experiencing predation. The total number of years of data collected is the entire duration of ‘Years of study’ unless otherwise noted in parenthesis. Span of data collected was through the late chick stage (L) or actual fledging (F); in some cases this information is unknown (U), or varied through the study (V). Some values are suspected to be biased low due to disturbance by researchers or other site visitors, as indicated.

| **Species** | **Colony location** | | **Years of study** | | **Breeding success** | **Span of data collection** | **Invasive predators present** | **Disturbance**  **suspected** | **Source** |
| --- | --- | --- | --- | --- | --- | --- | --- | --- | --- |
| ***Predators absent or controlled*** | | | | | | | | | |
| Cory's Shearwater  (*Calonectris diomedea*) | Chafarinas Islands, Spain | | 2000-2004 | | 0.58 | F | Black rat: controlled |  | [23] |
| Little shearwater  (*P. assimilis*) | Lady Alice Island, New Zealand | | 1995-1999 | | 0.68 | F | Koire (*R. exulans)*: controlled or absent |  | [24] |
|  | Coppermine Island, New Zealand | | 1997-1999 | | 0.56 | L | Koire: controlled or absent |  | [24] |
| Manx shearwater  (*P. puffinus*) | Skokholm Island, Wales | | 1973-1976 | | 0.70 | F | None reported |  | [21] |
|  | Rhum Isand, Scotland | | 1958-1985 (14) | | 0.58 | U | None reported |  | [25] |
|  | Canna Island, Scotland | | 1973-1985 | | 0.62 | U | None reported |  | [25] |
|  |  | |  | |  |  |  |  |  |
| Short-tailed shearwater  (*P. tenuirostris*) | Fisher Island, SE Australia | | 1950-1980 | | 0.49 | L | None reported | Yes | [9] |
| Sooty shearwater  (*P. griseus*) | Tuhawaiki Island | | unknown (3) | | 0.63 | U | None reported |  | [11] |
| Wedge-tailed shearwater (*P. pacificus*) | Muttonbird Island, Australia | | 1977-1979 | | 0.43 | L | None reported | Yes | [26] |
| ***Rattus present*** | | | | | | | | | |
| Audubon’s shearwater  (*P. lherminieri*) | | Santa Cruz Island, Galapagos, Ecuador | | 1965-1967 | 0.22 |  | Black rat: presumed | Possible | [27] |

| **Flesh-footed shearwater (*P. carneipes*)** | **Woody Island, Western Australia** | **2001-2002** | **0.47** | **F** | **Black rat** |  | **[18]** |
| --- | --- | --- | --- | --- | --- | --- | --- |
|  | **Lord Howe Island** | **2002** | **0.51** | **L** | **Black rat** |  | **[12]** |
| Little shearwater | Lady Alice Island, New Zealand | 1993-1994 | 0.43 | L | Koire (*R. exulans*) |  | [24] |
|  | Coppermine Island, New Zealand | 1995-1996 | 0.05 | L | Koire |  | [24] |
| Yelkouan shearwater  *(P. yelkouan*) | Port-Cros and Porquerolles Islands, France | 2003-2006 | 0.67 | U | Black rat, feral cat |  | [28] |
| ***Other predators present*** | | | | | | | |
| Black-vented shearwater (*P. opisthomelas*) | Natividad Island, Mexico | 1997-1998 | 0.36 | V | Feral cat |  | [29] |
| Hutton's shearwater  (*P. huttoni*) | South Island, New Zealand | 1989-1998 | 0.47 | L | Stoat (*Mustela erminea*) |  | [11] |
| Wedge-tailed shearwater | Kauai Island, Hawai'i, USA | 1978-1981 | 0.69 | F | Common myna (*Acridotheres tristis*) | Yes | [20] |

**Table 3.** Demographic matrices for flesh footed shearwaters on Lord Howe Island under four scenarios based on models described in Wilcox and Donlan [1] and corrected models described in this study; also given are asymptotic growth rates (λ) and percent increase in λ relative to those of the status quo scenario.

| **Scenario** | **W&D model (Model 1)** | **λ** | **% increase** | **Corrected model (Model 2)** | **λ** | **% increase** |
| --- | --- | --- | --- | --- | --- | --- |
| Status quo |  | 0.906 | 0 |  | 0.913 | 0 |
|  | *F_Ad_* = 0.50.7510.3730.8750.96 |  |  | *F_Ad_* = 0.50.7510.4340.8420.888 |  |  |
| Eliminate terrestrial impacts |  | 0.966 | 6.6 |  | 0.944 | 3.5 |
|  | *F_Ad_* = 0.50.7511 0.8750.96 |  |  | *F_Ad_* = 0.50.7510.7480.8420.888 |  |  |
| Eliminate bycatch |  | 0.986 | 8.8 |  | 0.992 | 8.6 |
|  | *F_Ad_* = 0.50.7510.3730.9750.96 |  |  | *F_Ad_* = 0.50.7510.4340.9500.888 |  |  |
| Eliminate terrestrial impacts and bycatch |  | 1.036 | 14.3 |  | 1.018 | 11.5 |
|  | *F_Ad_* = 0.50.75110.9750.96 |  |  | *F_Ad_* = 0.50.7510.7480.9500.888 |  |  |
